# Do uterine PTGS2, PGFS, and PTGFR expression play a role in canine uterine inertia?

**DOI:** 10.1007/s00441-021-03427-6

**Published:** 2021-04-08

**Authors:** Lea Magdalena Rempel, Karina Tietgen Andresen Lillevang, Ann-Kirstine thor Straten, Sólrún Barbara Friðriksdóttir, Hanna Körber, Axel Wehrend, Mariusz P. Kowalewski, Iris Margaret Reichler, Orsolya Balogh, Sandra Goericke-Pesch

**Affiliations:** 1 Reproductive Unit of the Clinics-Clinic for Small Animals, University of Veterinary Medicine Hannover, Foundation, Hannover, Germany; 2 Department of Veterinary Clinical Sciences, Section of Veterinary Reproduction and Obstetrics, University of Copenhagen, Taastrup, Denmark; 3 Klinikum Veterinärmedizin, Clinic for Obstetrics, Gynaecology and Andrology for Large and Small Animals with Veterinary Ambulance, Justus-Liebig-University Giessen, Giessen, Germany; 4 Institute of Veterinary Anatomy, Vetsuisse Faculty, University of Zurich, Zurich, Switzerland; 5 Clinic of Reproductive Medicine, Vetsuisse-Faculty, University of Zurich, Zurich, Switzerland; 6 Department of Small Animal Clinical Sciences, Virginia-Maryland College of Veterinary Medicine, Blacksburg, VA USA

**Keywords:** Dog, Prostaglandin, Dystocia, Uterine inertia, Placenta

## Abstract

The aetiology of primary uterine inertia (PUI), which is the most common cause of canine dystocia, is still not elucidated. Prostaglandins (PGs) play a crucial role in parturition. We hypothesized that the expression of prostaglandin endoperoxidase synthase 2 (PTGS2), PGF2α synthase (PGFS), and corresponding receptor (PTGFR) is altered in PUI. We investigated *PTGS2*, *PGFS*, and *PTGFR* mRNA expression, and PTGS2 and PGFS protein expression in interplacental (IP) and uteroplacental sites (UP) in bitches with PUI, obstructive dystocia (OD), and prepartum (PC). *PTGS2*, *PGFS*, and *PTGFR* mRNA expression did not differ significantly between PUI and OD (IP/UP). *PTGFR* ratio in UP was higher in PC than in OD (*p* = 0.014). PTGS2 immunopositivity was noted in foetal trophoblasts, luminal and superficial glandular epithelial cells, smooth muscle cells of both myometrial layers, and weakly and sporadically in deep uterine glands. PGFS was localized in luminal epithelial cells and in the epithelium of superficial uterine glands. PTGS2 and PGFS staining was similar between PUI and OD, while PGFS protein expression differed between OD and PC (*p* = 0.0215). For PTGS2, the longitudinal myometrial layer of IP stained significantly stronger than the circular layer, independent of groups. These results do not support a role for PTGS2, PGFS, and PTGFR in PUI. Reduced PGFS expression in IP during parturition compared with PC and the overall lack of placental PGFS expression confirm that PGFS is not the main source of prepartal PGF2alpha increase. The difference in PTGS2 expression between IP myometrial layers warrants further investigation into its physiological relevance.

## Introduction

Uterine inertia is the most common cause of dystocia in the parturient bitch accounting for 54.7–70% of all dystocia cases (Darvelid and Linde-Forsberg 1994; Gaudet 1985). It may negatively affect maternal as well as foetal health and survival, if not recognized and treated in time. Medical treatment of uterine inertia has a relatively low success rate (Münnich and Küchenmeister 2009). In a study, 54.3% of the treated bitches failed to respond to the treatment with oxytocin and/or denaverine (Münnich and Küchenmeister 2009). This is one of the reasons why affected bitches often directly undergo emergency Caesarean section (C-section) (Bergström et al. 2006a; Darvelid and Linde-Forsberg 1994; Prashantkumar and Walikar 2018). Although there is good agreement about how to manage this clinical condition, the aetiology of uterine inertia, the failure of functional myometrial contractions, is far from being understood. It is likely to be of multifactorial origin caused by defects at both cellular and molecular levels (Davidson 2011; Münnich and Küchenmeister 2009).

Previous studies on canine uterine inertia investigated the role of progesterone (P4) and suspected failure of luteolysis (Bergstrom et al. 2010; Irons et al. 1997; McLean 2012; van der Weyden et al. 1989), a possible role of parathyroid hormone (PTH) (Hollinshead et al. 2010), and the availability of ionized calcium or glucose during parturition (Hollinshead et al. 2010). Other causes have also been postulated to predispose to uterine inertia such as systemic disease of the bitch, small litter size (e.g., single-pup syndrome), overstretching of the myometrium due to a large litter or excessive foetal fluids, obesity and fatty infiltration of the myometrium, hypocalcaemia, hypoglycaemia, nutritional imbalance, age-related changes, or genetic inheritance (Frehner et al. 2018; Kraus and Schwab 1990; Linde Forsberg and Eneroth 2000; Linde Forsberg and Persson 2007; Simões et al. 2016).

As the aetiology is still unclear, we studied changes occurring at the molecular level of the uterus to explain contractile inability in primary uterine inertia (PUI). Our group recently demonstrated significant differences in uterine smooth muscle γ-actin and smooth muscle myosin gene expression between PUI bitches and dogs with strong labour contractions during obstructive dystocia (Egloff et al. 2020), while another research group found no association between uterine oxytocin receptor expression and PUI in dogs (Tamminen et al. 2019). These results are encouraging and indicate that further studies are needed to identify the respective cellular and molecular changes in the uterus implicated in the development of PUI.

Throughout pregnancy, the uterus undergoes substantial changes in order to fulfil different demands. First, the myometrium has to be in a stage of quiescence, which is mediated by luteal progesterone in the dog (Concannon et al. 1989; Verstegen-Onclin and Verstegen 2008). Before parturition, however, it is essential that the myometrium is activated to become a “contractile organ.” This change is associated with increased expression of contraction-associated proteins, such as prostaglandin endoperoxidase-2 (PTGS2, previously known as cyclooxygenase 2, COX2); receptors for prostaglandins (PGs), such as the PGF2α receptor (PTGFR); and oxytocin (OT) receptor, gap junction proteins, and proteins encoding ion channels as reviewed for various species, such as ewe, cow, sow, dog, and human (Challis et al. 2002; Cook et al. 2000; Gram et al. 2013; Gram et al. 2014; Kowalewski et al. 2010; Norwitz et al. 1999; Olson 2003; Patel and Challis 2001).

Kowalewski et al. (2010, 2012) have previously shown that the well-known significant increase of PGF2α that induces prepartum luteolysis and thereby initiates the parturition process (Baan et al. 2008; Baan et al. 2005; Concannon and Hansel 1977; Concannon et al. 1988; Concannon et al. 1989; Olsson et al. 2003) is associated with a strong increase in PTGS2 mRNA and protein expression in the foetal trophoblasts (Gram et al. 2013; Gram et al. 2014; Kowalewski 2012; Kowalewski et al. 2010). PGs are also known to be critically involved in the stimulation of myometrial contractions in sheep (Whittle et al. 2000), in cattle (suffering from retained foetal membranes (Takagi et al. 2002)), and in humans (Patel and Challis 2001). The role of placental or locally derived PGs as uterotonic agents on the myometrium has also been postulated in the dog (Hoffmann et al. 1999; Nohr et al. 1993) and molecular PGE2/PGF2α pathway analysis of bitches undergoing elective C-section during prepartal luteolysis support this (Kowalewski et al. 2010; Nowak et al. 2019).

PTGS2, the key enzyme and rate-limiting factor for prepartal PG synthesis (Kowalewski et al. 2010; Kowalewski et al. 2014; Wiltbank and Ottobre 2003), plays a central role in parturition. The canine PGF2α synthase (PGFS), also known as aldo-keto reductase family member 1 (AKR1C3), is the enzyme responsible for reduction of prostaglandin endoperoxide H2 (PGH2) to PGF2α. PGF2α is, together with PGE2, involved in coordination of functional myometrial contractions (Challis et al. 2002; Gram et al. 2013). Additionally, in the dog PGE2 can be actively utilized for the synthesis of PGF2α and the respective synthetic activity is higher at prepartum (Gram et al. 2014). Consequently, it seems reasonable that altered PTGS2, PGFS, or PTGFR mRNA and/or protein expression, function, or localization might lead to uterine inertia. Neither PTGS2 expression nor expression of other prostaglandin synthases and receptors has been investigated yet during normal canine parturition comparing it to uterine inertia. Therefore, the aim of our study was to investigate PTGS2, PGFS, and PTGFR mRNA and protein expression in interplacental and placentation sites of the uterus in bitches diagnosed with PUI and to compare it with the expression in bitches undergoing C-section because of obstructive dystocia (OD). Furthermore, uterine samples obtained from planned C-sections (PC) before prepartum luteolysis and from OD after luteolysis seem to be suitable to characterize changes occurring during the periparturient period.

## Material and methods

Animal experimentation was approved by the respective authorities (permit no. 2015-15-0201-00513, Dyreforsøgstilsynet Fødevarestyrelsen; permit no. ZH086/15, Cantonal Veterinary Office Zurich).

### Animals and study design

Twenty clinically healthy bitches at term pregnancy presented at the respective clinics for management of parturition or dystocia were included in this study. C-section was performed only if it was medically indicated. Reasons for C-section were primary uterine inertia (*n* = 10) and obstructive dystocia (OD, *n* = 5) (for definitions and grouping see 2.3). Additionally, five bitches undergoing planned C-sections were included. Mean body weight of the bitches was 20.02 ± 16.42 kg (range: 3.6–70.1 kg) and mean age 3.93 ± 2.07 years (range 0.9–8.1 years). Details about the included dogs are given in Table 1. All bitches underwent a general physical and obstetrical examination, including blood sampling for blood cell counts, determination of ionized calcium and progesterone by RIA (Hoffmann et al. 1992; Hoffmann et al. 1994; Hoffmann et al. 1973), tocodynamometry (for 20 min, but only if the bitch was stable and the foetal heart rates were not reduced, namely > 200 bpm), and abdominal ultrasonography (to monitor foetal viability). To diagnose obstructive dystocia due to foetal malpresentation/-position/-posture or foetal oversize/malformation, abdominal/pelvic x-rays (laterolateral and dorsoventral) were performed if digital vaginal examination could not rule out obstruction.

**Table 1:** Breed, age, bodyweight, body condition score (BCS), and current pregnancy number of the bitches. Bitches were grouped into primary uterine inertia (PUI), obstructive dystocia (OD), and planned C-section before birth (PC). Details about collected samples (interplacental, IP, and uteroplacental, UP, tissue) and use for mRNA analysis and protein localization by immunohistochemistry (IHC) are provided

Group	Breed	Age(years)	Body weight (kg)	Body condition score	Current pregnancy number	Litter size	mRNAanalysis		IHC	
							IP	UP	IP	UP
PUI (*n* = 10)	Dachshund	6.5	6.9	6	2	2	X	-	X	-
	Boxer	5.2	29	6	1	2	X	-	X	-
	French Bulldog	1.9	-	5	2	2	X	-	X	-
	Broholmer	4.1	70.1	4	2	7	X	-	X	-
	German Shepherd	7.1	34.1	5	4	7	X	-	X	-
	Labrador	7	36	6	3	6	X	X	X	X
	Maremmano	5	40	3	-	11	X	-	X	-
	Maltese	2	7.25	4	1	8	X	X	X	X
	Beagle	4.1	19.7	5	2	9	X	X	X	X
	Kooikerhondje	2.1	13	3	1	11	X	-	X	-
OD (*n* = 5)	Cairn Terrier	3.8	11.1	5	3	6	X	-	X	-
	Staffordshire Bullterrier	2.1	12.5	3	1	4	X	-	X	-
	Mongrel	0.9	21.2	5	1	6	X	X	-	-
	WHWT	8.1	10.3	4	2	3	X	X	X	X
	Yorkshire Terrier	3.6	3.6	5	1	3	X	X	X	-
PC (*n* = 5)	WHWT	3	11.5	5	2	4	X	X	X	X
	Labradoodle	4.2	27	4	3	1	X	X	X	-
	French Bulldog	1.4	9	5	1	2	X	-	X	-
	Chihuahua	4.5	4.5	4	3	6	X	X	-	X
	French Bulldog	2	13.6	6	3	2	X	X	X	X

*X* sample available, - sample not available

### Tissue sample collection and processing

After delivery of all puppies during C-section, full thickness uterine samples were collected from the incision site (interplacental site, IP) in the uterine horn. The IP samples had a width of about 1 cm and the length of the uterine incision necessary for removal of the puppy/puppies during C-section. In cases where bitches were ovariohysterectomized, additional full thickness samples including the placenta were taken from uteroplacental sites (UP). For the preservation of tissue for RNA and protein extraction, full thickness samples from IP or UP were immersed in RNAlater^®^ (Ambion Biotechnologie, Wiesbaden, Germany) for a minimum of 24 h at 4 °C and then stored at − 80 °C. Tissue samples for immunohistochemistry (IHC) were fixed in 10% neutral phosphate-buffered formalin at 4 °C for 24 h and washed several times in phosphate-buffered saline before dehydration in ethanol series and embedding in paraffin.

### Grouping of dogs

All dogs were assigned to one of the respective groups based on history and clinical findings as follows (Frehner et al. 2018):

- Group 1: Primary uterine inertia (PUI; *n* = 10), defined as bitches in birth that failed to give birth to any puppies. Additionally, at least one of the following criteria had to be present in the history of the bitch and/or during obstetrical examination: Signs of first stage labour and/or prepartum temperature drop ≥ 20 h earlier or the body temperature was normalized again, but no progress to second stage labour; no signs of abdominal contractions, but foetal fluids passed > 3 h and no progression since then; no other signs of second stage labour, but green vulvar discharge for > 2 h; infrequent, weak, unproductive abdominal contractions for more than 4 h without progress; and the Ferguson reflex could not or only weakly be triggered by digital stimulation per vagina (Johnston et al. 2001; Linde-Forsberg 2010). Tocodynamometry revealed only weak or no uterine contractions, and obstruction could be ruled out (digital vaginal examination and/or abdominal radiographs).
- Group 2: Obstructive dystocia (OD; *n* = 5) was diagnosed by digital vaginal palpation and/or radiographs. These bitches were in second stage labour still showing strong spontaneous abdominal and/or uterine contractions as they were actively trying to deliver the pups, and showed strong straining after vaginal digital manipulation (feathering). These clinical findings were the opposite of the PUI group, and therefore, these bitches were considered well-suited for comparison of uterine contractile function.
- Group 3: Planned C-section before birth (PC; *n* = 5), defined as bitches undergoing planned C-section due to e.g. brachycephalic or achondroplastic breed and high risk of dystocia. Bitches had no history of uterine inertia. At the time of C-section, serum progesterone was > 2 ng/ml.

Grouping was done retrospectively for groups 1 and 2, and prospectively for group 3. Information about all dogs is given in Table 1.

### Real-time PCR

Total RNA from RNAlater^®^ immersed tissue samples was isolated using Trizol^®^ (Life Technologies, Darmstadt, Germany) according to the manufacturer’s protocol. RNA concentration and quality were assessed using a spectrophotometer (NanoDrop^®^ ND-1000, NanoDrop Technologies, Wilmington, USA). Full-length first strand cDNA synthesis was carried out using 200 ng/μl RNA per sample and the RevertAidFirst Strand cDNA Synthesis Kit (#K1622, Thermo Scientific, Waltham, MA, USA) according to the manufacturer’s protocol (including DNAse treatment).

To test for expression of *PTGS2* (for: GGAGCATAACAGAGTGTGTGATGTG, rev: AAGTATTAGCCTGCTCGTCTGGAAT, amplicon length: 228bp, efficiency: 2.17, accession number: NM_001003142), *PGFS* (for: AAGGACCCAGTTCTCAATGC, rev: AGTTCTCCCGGATTCTCTTC, amplicon length: 133bp, efficiency: 2.01, accession number: NM_001012344), and *PTGFR* (for: CAGTGCCCTGGTAATCACAG, rev: GCGGATCCAGTCTTTATCGG, amplicon length: 91bp, efficiency: 2.15, accession number: NM_ 001048097), quantitative real time PCR (RT-qPCR) was used as previously described for the canine testis (Körber and Goericke-Pesch 2018). RT-qPCR was performed by adding 2 μl cDNA (dilution 1:10) to 5 μl iQTM SYBR Green Supermix (Roche Diagnostics, Basel, Switzerland), 1 μl of the forward and reverse primer (10 pmol), and 1 μl sterile Aqua bidest. RT-qPCR conditions were identical to our previously published protocol (Körber and Goericke-Pesch 2018): 95 °C for 5 min, 40 cycles of 95 °C for 10 s, 60 °C for 10 s, 72 °C 20 s, and melting curve with 65–97 °C. *Glyceraldehyde-3-phosphate dehydrogenase *(*GAPDH*, for: GGCCAAGAGGGTCATCATCTC, rev: GGGGCCGTCCACGGTCTTCT, amplicon length: 88bp, efficiency: 1.97, accession number: NM_001003354) and *ß-actin* (for: GCTGTGCTGTCCCTGTATG, rev: GCGTACCCCTCATAGATGG; amplicon length: 98bp, efficiency: 2.1, accession number: AF484115.1) were chosen as reference genes (Bustin et al. 2009), but as *GAPDH* was most stable, it was used for data analysis. All samples were run in triplicates using a LightCycler^®^480 real-time PCR system (Software release 1.5.0, Version 1.5.0.39, Roche), and a non-template control was included in each assay. PCR efficiencies of target and reference genes were calculated with the Roche Light Cycler® 480 SW 1.5 software by using a relative standard curve derived from a triplet RT-qPCR run of a 2-fold dilution series (1:2 – 1:128) of pooled cDNA samples, where the efficiency (*E*) was: *E* = 10(− 1/m) with m being the slope of the linear regression line (Pfaffl 2001). Evaluation of the RT-qPCR results was an efficiency-corrected relative quantification according to Pfaffl (Pfaffl 2001).

The specificity of the *PTGS2*, *PGFS*, and *PTGFR* primers was checked using BLAST (http://blast.ncbi.nlm.nih.gov), and results were confirmed by sequencing of PCR products (Beckman Coulter Genomics, United Kingdom). All primers were synthesized by TAG Copenhagen A/S.

### Immunohistochemistry for protein expression of PTGS2 and PGFS

Immunohistochemistry was only performed for PTGS2 and PGFS due to the lack of a commercially available canine PTGFR antibody. Sections of 3 μm were cut from the paraffin embedded tissue samples and mounted on SuperFrost Plus microscope slides (Thermo Scientific/Menzel-Gläser, Braunschweig, Germany). The same primary antibodies against PTGS2 (monoclonal mouse anti-rat PTGS2 antibody, clone 33/COX-2, BD Pharmingen, Heidelberg, Germany) and PGFS (canine-specific custom-made polyclonal, affinity-purified guinea pig anti-PGFS (AKR1C3), Eurogentec Seraing, Belgium) were used as published earlier and found to be specific in canine IP and UP tissue (Kautz et al. 2014; Kowalewski et al. 2010; Kowalewski et al. 2014). Following deparaffinization and rehydration, an immunoperoxidase method was applied as previously described (Körber and Goericke-Pesch 2018) using the above described primary antibodies (PTGS2 antibody, dilution 1:100 corresponding to 1.25 µg/mL; PGFS antibody, dilution 1:750) and a biotinylated horse anti-mouse IgG (BA-2000, Vector Laboratories, dilution 1:200, 0.0075 µg/ml). The secondary antibodies were a biotinylated horse anti-mouse IgG (BA-2000, Vector Laboratories, dilution 1:200, 0.0075 µg/ml for PTGS2), and a biotinylated goat anti-guinea pig antibody (BA-7000, Vector Laboratories, dilution 1:100, 2 µg/ml for PGFS) diluted in 10% horse (PTGS2) or goat serum (PGFS). Negative controls were performed using irrelevant isotype controls at the same protein concentration (PTGS2: Mouse IgG I, clone W3/25; PGFS: Guinea Pig IgG I-7000, LINARIS Biologische Produkte GmbH, Dossenheim, Germany) and depletion of the primary antibody. Canine ovarian tissue including corpora lutea was used as positive control for PTGS2 (Kowalewski et al. 2006).

Evaluation of the PTGS2 staining was performed by two independent researchers, whereas PGFS was evaluated by a single observer using an BX41TF light microscope (Olympus Europa SE & Co. KG, Hamburg, Germany). All researchers were blinded to the groups. For each dog, the localization of the immunopositive signals indicating the presence of PTGS2 or PGFS protein was assessed. Furthermore, a detailed evaluation of positively stained cell types was performed for each dog evaluating the staining intensity in the longitudinal and circular muscle layers by a predefined scale (ordinal score system; 0: no; 1: weak, 2: strong, 3: very strong). For PTGS2, the longitudinal and circular myometrial muscle layers of the IP and UP samples were evaluated, taking the staining of the cytoplasm and the cytoplasmic granules of the smooth muscle cells individually into consideration. In case of PGFS, a detailed evaluation of the cytoplasmatic and nuclear staining intensity in the uterine luminal epithelial cells and the glandular epithelium of the superficial uterine glands in the IP and UP samples (evaluated together) was done following the immunoreactive score (IRS) method, a method well-known for evaluation of heterogenic IHC samples (Fedchenko and Reifenrath 2014). Multiplying the signal intensity with its proportion and then summarizing the categories have been shown to give a more accurate result (Specht et al. 2015); therefore, the multiplication with summation method was used here.

### Statistical analysis

For all statistical analysis, Microsoft Excel (Windows XP; Microsoft) and the statistical software program Graph Pad Prism7 (GraphPad Software, Inc., La Jolla, USA) were used. Values were statistically significant at a level of *p* ≤ 0.05.

The primary overall aim was to identify changes due to PUI by comparison of PUI and OD samples. Furthermore, we aimed to identify changes in the periparturient period (before and after luteolysis) comparing PC and OD samples. Significant differences in *PTGS2*, *PGFS*, and *PGTFR* mRNA or PTGS2 and PGFS protein expression were investigated between groups (PUI versus OD and PC versus OD, individually for IP and UP each). All datasets were initially tested for normal distribution by Shapiro-Wilk test before further analysis was conducted.

Logarithmic transformation of *PTGS2* and *PGFS* mRNA expression (ratio) data revealed normal distribution. An unpaired *t* test was applied to test for significant differences between PUI and OD or PC and OD. For *PTGFR*, all data except PC versus OD (UP) was also normally distributed, so in this one case, the Mann-Whitney test was used to identify significant differences between the groups.

PTGS2 protein expression was descriptive followed by more detailed analysis of the myometrial staining. The myometrium was selected for further evaluation as it exhibited the most pronounced staining intensity (see “Results”). All these individual results obtained from both independent evaluators were compared for inter-evaluator agreement using the weighted kappa (K) coefficient on raw data (Jakobsson and Westergren 2005). As the inter-evaluator agreement was found to be “very good” with the weighted K coefficient being 83.8, the mean values of both evaluators were used for all further analysis resulting in individual “staining means” of the cytoplasm and the granules of the longitudinal and the circular muscle layer for each sample, respectively.

To test for significant differences between the respective groups regarding the scoring intensity of the PTGS2 protein, an overall myometrial staining score (OMSS) was calculated for each dog. Due to group size, these calculations were only performed for IP, but not for UP samples. To calculate the OMSS, initially an individual score of the longitudinal and the circular myometrial layer was calculated based on the mean of the scores of the cytoplasmatic staining and the granular staining of the respective layer. These scores (mean longitudinal/circular score) were used to calculate the mean staining intensity of the circular and longitudinal muscle layer with OMSS = ((mean longitudinal score + mean circular score)/2). The myometrial staining intensity (OMSS) in IP tissue samples was compared between PUI and OD samples to identify possible differences due to uterine inertia as well as between OD and PC to identify differences between before and in birth using the Mann-Whitney test. To test for differences between staining intensity between the longitudinal and circular muscle layer of the IP samples in dogs in birth, data of all PUI and OD bitches were summarized and analysed using the Wilcoxon signed rank test.

Due to the tissue localization pattern of the PGFS protein (see “Results”), endometrial staining intensity was evaluated in IP and UP. Endometrial “staining means” were derived from cytoplasmatic and nuclear staining (nuclear staining intensity + cytoplasmatic staining intensity/2) and used for all further statistical calculations. Following the Shapiro-Wilk test, the endometrial staining intensity was compared in both IP and UP tissues between PUI and OD to identify possible differences due to uterine inertia as well as between OD and PC to identify differences between before and in birth using an unpaired t-test.

## Results

### *PTGS2*, *PGFS*, and *PTGFR* mRNA expression

**Fig. 1:**
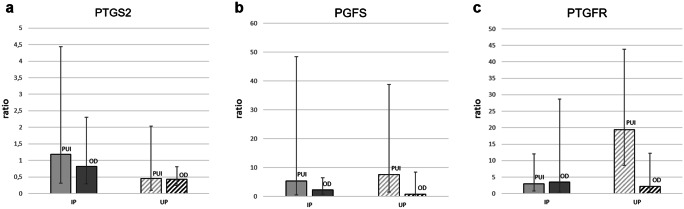
Relative gene expression (ratio, x̅g (DF)) of **a** *prostaglandinendoperoxidase-2* (*PTGS2*), **b** *prostaglandin F2*α synthase (*PGFS*), and **c** *prostaglandin F2α receptor* (*PTGFR*) in interplacental (IP) and uteroplacental (UP) tissue homogenates comparing samples from bitches diagnosed with primary uterine inertia (PUI) or obstructive dystocia (OD)

**Fig. 2:**
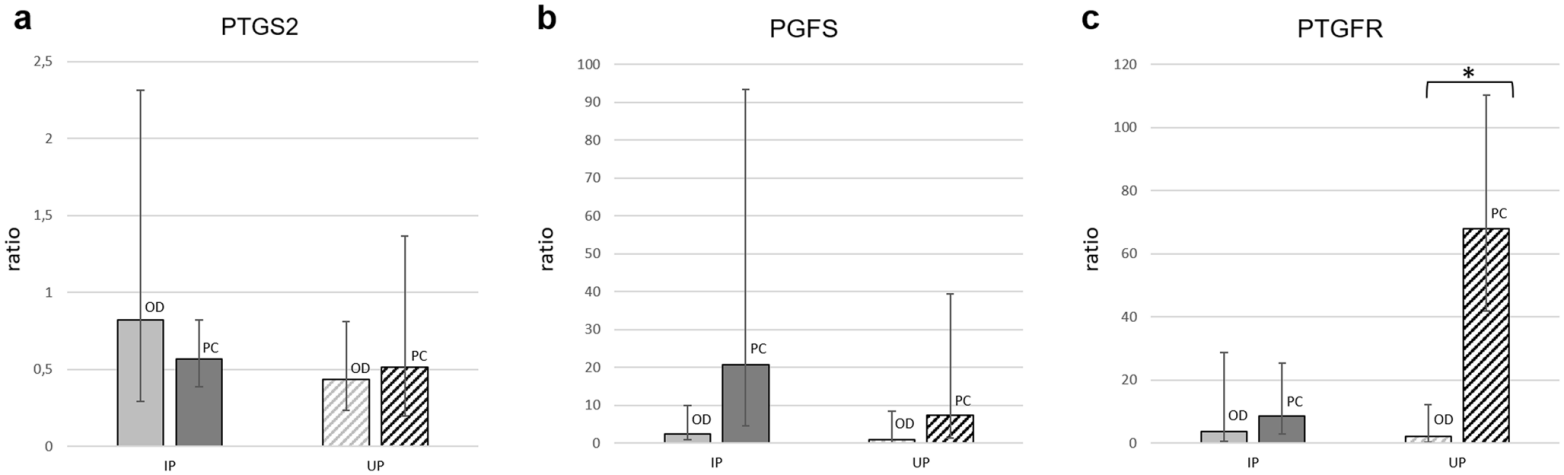
Relative gene expression (ratio, x̅g (DF)) of **a** *prostaglandinendoperoxidase-2* (*PTGS2*), **b** *prostaglandin F2α* *synthase* (*PGFS*), and **c** *prostaglandin F2α receptor* (*PTGFR*) in interplacental (IP) and uteroplacental (UP) tissue homogenates of bitches diagnosed with obstructive dystocia (OD) or presented for planned C-section before birth (PC). * indicates a significant difference (*p* ≤ 0.01) between relative *PTGFR* gene expression in UP tissue homogenates of bitches with OD and PC

*PTGS2*, *PGFS*, and *PTGFR* mRNA expression (ratio) did not differ significantly (*p* > 0.05) between PUI and OD neither in IP nor in UP tissue samples (Fig. 1). Also, no significant differences (*p* > 0.05) were found comparing *PTGS2* and *PGFS* ratios between OD and PC in IP and UP tissue samples (Fig. 2a, b). *PTGFR* mRNA expression (ratio), however, was significantly increased in PC compared with OD in UP tissue samples (*p* = 0.014), whereas no difference was found in IP tissue samples (*p* > 0.05) (Fig. 2c).

### PTGS2 and PGFS protein expression

**Fig. 3:**
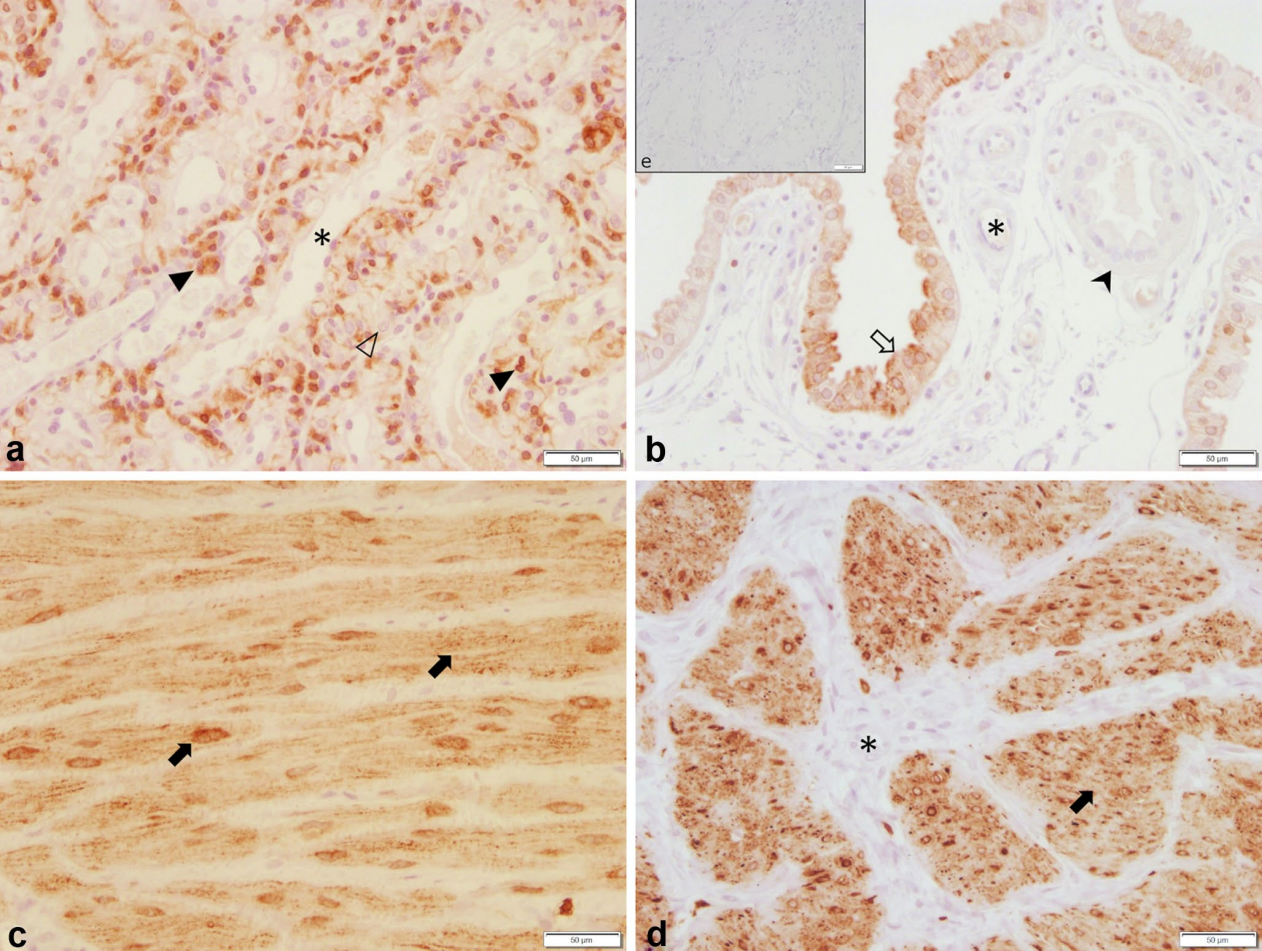
PTGS2 protein localization as revealed by immunohistochemical (IHC) staining in canine uterine tissue (**a** uteroplacenta, UP; **b**–**e** interplacenta, IP). **a** Placenta, **b** uterine luminal epithelium and superficial uterine glands, **c** and **d** myometrium (**c** Stratum circulare, **d** Stratum longitudinale), and **e** isotype control for PTGS2 given as insert. Specific cell types are indicated with symbols: (➪), uterine luminal epithetal cells, (➤) = glandular epithelium of superficial uterine glands, (➨) = myocites, (△) = maternal decidual cells, (▲) = fetal trophoblast cells, (*) = blood vessels

Immunohistochemistry revealed a regular specific immunostaining for PTGS2 and PGFS in all samples investigated. The specific staining against PTGS2 was observed in foetal trophoblast cells of the placental labyrinth (Fig. 3a), endometrial epithelial cells (Fig. 3b), and smooth muscle cells of both myometrial layers (Fig. 3c, d) with the myocytes staining strongest compared with all other immunopositive cells. Additionally, some deep uterine glands stained weakly (not shown). Interestingly, trophoblast cell staining intensity and distribution varied between animals with a strong, uniform staining of the entire trophoblast in the OD UP sample (*n* = 1) and an incomplete/inhomogeneous staining of the trophoblast in PUI UP samples (*n* = 3). Due to the low number of UP samples, no statistical analysis was performed.

**Fig. 4:**
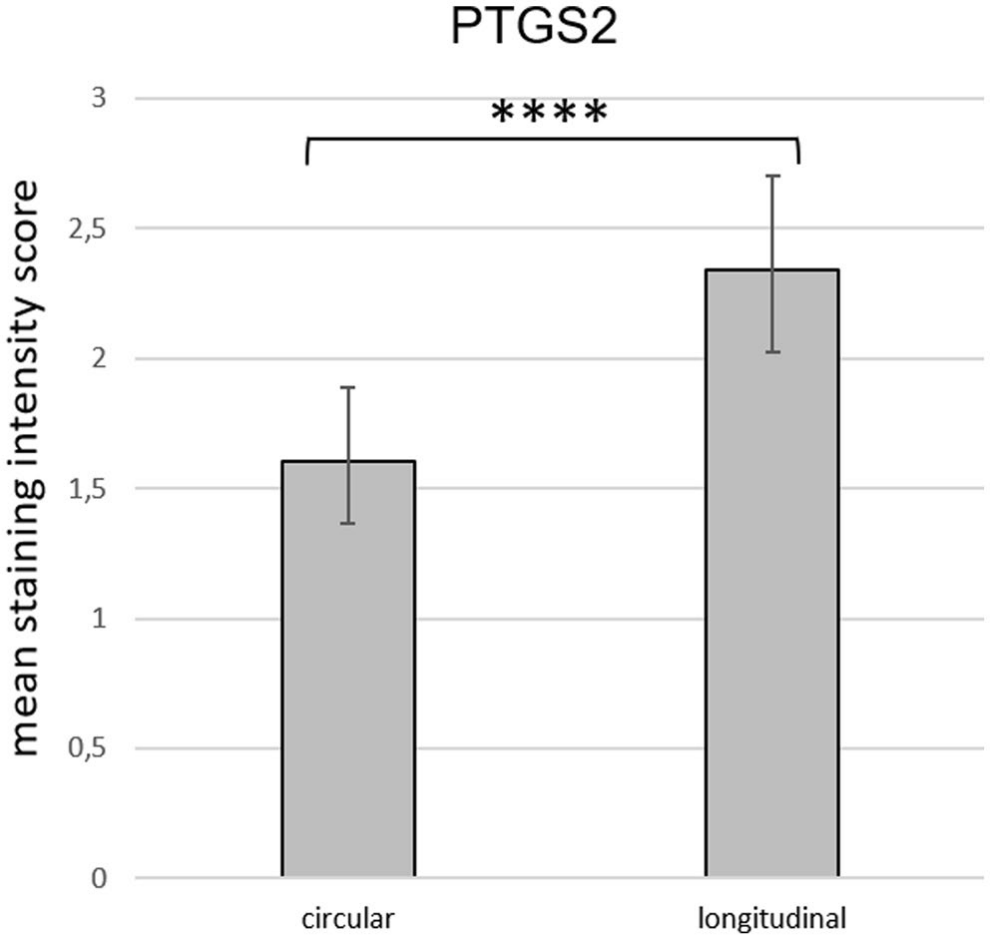
Mean PTGS2 staining intensity score as revealed by immunohistochemistry in the two myometrial layers (circular versus longitudinal) of interplacental tissue from bitches in labour (primary uterine inertia, PUI, and obstructive dystocia, OD, combined) **** indicates a significant difference between the mean staining scores of the circular and the longitudinal uterine muscle layer (*p* ≤ 0.0001)

Subjectively, PTGS2 staining intensity of endometrial cells and uterine glands did not differ between groups and sample localization. Overall myometrial staining score (OMSS), representing myometrial PTGS2 protein expression (IP samples only), did not differ between experimental groups (OD vs PUI or PC). Because signal intensity seemed to differ between the longitudinal and circular myometrial layer in IP samples, systematic scoring was performed and analysed statistically. Comparing PTGS2 expression between these two myometrial layers of the IP in all bitches in birth (PUI and OD summarized), the staining intensity was significantly higher in the longitudinal compared with the circular myometrial layer (*p* = 0.0001) (Fig. 4).

**Fig. 5:**
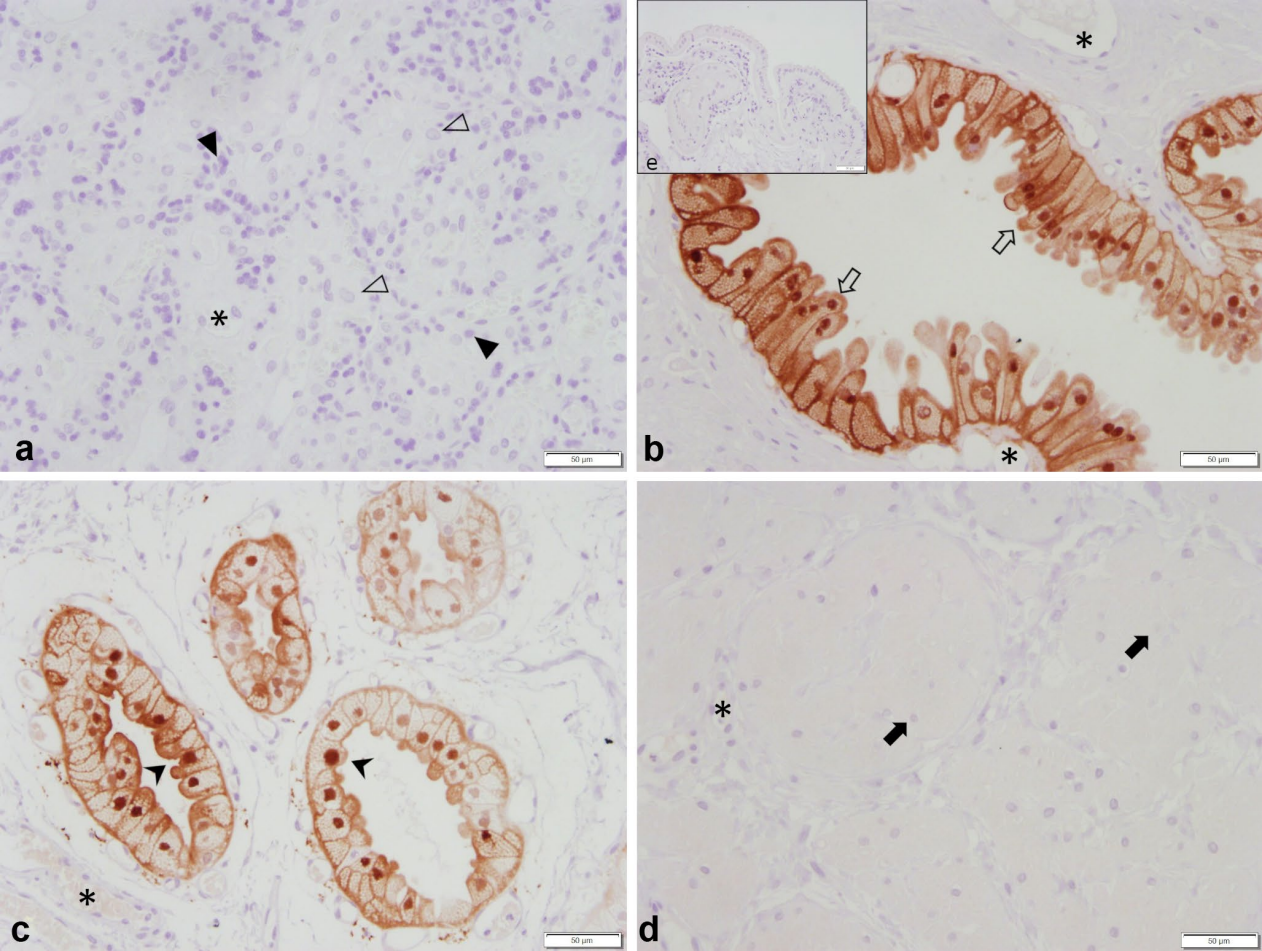
PGFS protein localization as revealed by immunohistochemical (IHC) staining in canine uteroplacental tissue (**a**+**b**: uteroplacenta, UP; **c**–**e**: interplacenta, IP). **a** Placenta, **b** epithelial cells of the glandular chambers, **c** superficial uterine glands, **d** myometrium, and **e** isotype control for PGFS given as an insert. Specific cell types are indicated with symbols: (➪) = uterine luminal epithetal cells, (➤) = glandular epithelium of superficial uterine glands, (➨) = myocites, (△) = maternal decidual cells, (▲) = fetal trophoblast cells, (*) = blood vessels

**Fig. 6:**
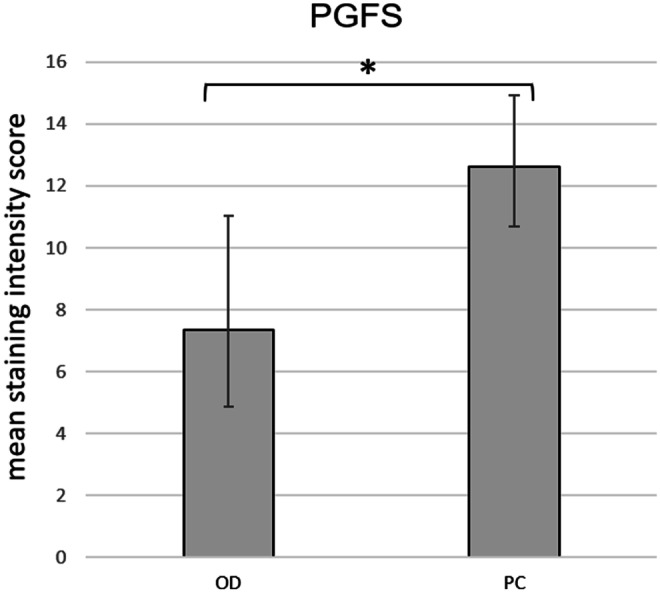
Mean endometrial PGFS staining intensity score as revealed by immunohistochemistry in canine interplacental tissues from bitches diagnosed with obstructive dystocia (OD) or bitches presented for planned C-section before birth (PC). * indicates significant difference (*p* ≤ 0.05) between the OD and PC groups

The UP expression of PGFS was localized in the uterine luminal epithelial cells (glandular chambers) (Fig. 5b) and the glandular epithelium of the superficial glands (Fig. 5c). No signal was detectable in the myometrial layers (Fig. 5d), in uterine stromal cells, or in the placental labyrinth (Fig. 5a) in any of the groups. In some dogs, a very weak signal was observed in the glandular epithelium of the deep uterine glands (not shown). The mean endometrial staining scores did not differ between PUI and OD, whereas a significant difference (*p* = 0.0215) was found between PC and OD in IP tissue samples (Fig. 6) with higher endometrial expression in the PC group.

## Discussion

Currently, our knowledge about the underlying aetiology of canine PUI is still very limited and most studies refer to hormones and metabolic blood parameters, such as progesterone, oxytocin, calcium, and glucose (Bergstrom et al. 2010; Bergström et al. 2006b; Davidson 2011; Frehner et al. 2018; Hollinshead et al. 2010; Kraus and Schwab 1990; Lucio et al. 2009).

PUI is characterized by inadequate/insufficient uterine contractions, and PTGS2 and prostaglandins play an essential role in the coordination of uterine contractions (Challis et al. 2002; Noakes et al. 2009, Senior et al. 1991; Senior et al. 1993; Senior et al. 1992). Consequently, the aim of this study was to identify the role of PTGS2, PGFS, and PTGFR in the aetiology of PUI. Collecting uterine/uteroplacental samples from eutocic bitches with normal, effective myometrial contractility is not possible in a clinical setting, and subjecting a bitch with normal labour progression to surgery only for research sample collections is of severe ethical concern and not appraised by the authors. In contrast, samples derived from bitches diagnosed with obstructive dystocia still showing strong labour contractions were available and therefore used for comparison. We have previously described OD as a control for comparison of uterine contractile proteins actin and myosin with PUI (Egloff et al. 2020), and Tamminen et al. (2019) also used the OD group for comparison of the uterine oxytocin receptor expression in dystocic bitches (Tamminen et al. 2019). It is without a doubt that OD is not equal to normal parturition as progress of parturition ceases due to obstruction, revealing not a physiological situation. However, based on the history, the detailed clinical examinations, the fact that all OD bitches showed strong straining in response to vaginal digital manipulation (feathering) as well as strong spontaneous abdominal and/or uterine contractions, we concluded that the dogs still had uterine contractions and did not reach uterine fatigue and secondary inertia yet. Even if digital vaginal manipulation is a spinal reflex and does not necessarily represent uterine contractions in all cases, they can be confirmed by tocodynamometry in some and are frequently used for decision-making of the clinician. Furthermore, three of the OD bitches gave birth to one or two puppies before obstruction indicating that uterine contractility had been adequate initially. The current literature about tocodynamometry patterns in OD bitches is scarce and controversial: Schröder (2008) and Davidson (2003a, b) reported significantly altered contraction patterns in case of OD, whereas Tamminen (2020) recently showed that mean number of contractions/10 min, change of amplitude % units (mean), interval between contractions, and durations of contractions did not differ between OD and normal parturition (Davidson 2003a, b; Schröder 2008; Tamminen 2020).

The PC group was considered to represent the uterine status before prepartal luteolysis, namely, the status before birth. As reviewed and investigated in detail, significant endocrine changes occur before birth, such as the prepartum luteolysis (Concannon 2011; Kowalewski 2012; Kowalewski et al. 2015; Kowalewski et al. 2020) as well as significant changes in placental gene expression. Although Kowaleweski and coworkers investigated in detail the changes during canine pregnancy, they focused on the comparison of early, mid pregnancy and prepartum luteolysis (Kowalewski et al. 2020; Nowak et al. 2019). To the best of our knowledge, uterine gene expressions have not been compared between tissues obtained from before and after prepartal luteolysis, with the latter being represented by OD in our study. Even if the C-section was planned because bitches were considered to have a high risk of dystocia, bitches had no history of PUI earlier. A limitation of this group is the small sample size and the fact that one of the bitches had a single-pup pregnancy.

Although *PTGS2*, *PGFS*, and *PTGFR* mRNA expression was verified in all samples as previously described in prepartal bitches in UP samples (Kowalewski et al. 2010; Kowalewski et al. 2014), other than hypothesized, no significant differences were identified between the PUI and OD groups (IP and UP). This indicates a questionable role for *PTGS2*, *PGFS*, and *PTGFR* expression in the aetiology of PUI. While no significant differences between OD and PC were identified for *PTGS2* and *PGFS* in IP and UP samples, our finding of different uteroplacental *PTGFR* expression between these groups suggest that in dogs, *PTGFR* expression is significantly modulated during the onset of parturition. It is plausible that as labour ensues and uterine contractions proceed, concomitant with large increases in peripheral PGFM concentrations (Baan et al. 2008; Baan et al. 2005; Concannon and Hansel 1977; Concannon et al. 1988; Concannon et al. 1989; Olsson et al. 2003), the sensitivity of the UP to PGF2α decreases. Whether downregulation of *PTGFR* expression in UP is primarily due to changes in the placental labyrinth rather than the endometrium or myometrium, it needs further verification.

In order to confirm the lack of significant differences in *PTGS2*, *PGFS*, and *PTGFR* mRNA expression, mRNA retrieval from individual uterine layers (placenta/endometrium/myometrium) and subsequent “layer-specific” mRNA expression analysis may be performed for comparison between groups and localizations. To what extent the small sample sizes (especially in UP samples) and heterogeneity of our groups in terms of breeds/body weight and litter size might have had an impact on the results remains to be clarified. In our studies on PUI (Egloff et al. 2020); Frehner et al., submitted) using very similar groups of dogs, the bitches’ body weight was found to have no effect on IP gene expression. Although breed differences may be considered, body weight is probably a better indicator of dog size variability. The heterogeneity of samples included in this study is representative for the existing breeding population and reflects “real-life situation.”

PTGS2 protein expression was identified in all groups within foetal trophoblast cells (UP only), luminal epithelial cells and uterine glands in the endometrium, and myocytes of both myometrial layers. This localization pattern found in our tissue samples is in good agreement with previous studies on the pregnant canine uterus (Kowalewski et al. 2010; Kowalewski et al. 2014). Interestingly, some variation between animals and groups was observed in foetal trophoblast cell staining in a relatively small sample size. Recently, the comparative functions of placental trophoblast cells had been reviewed in different animal species, such as ruminants, pig, horse, dog, and cat (Peter et al. 2017). This review clearly summarizes our still restricted understanding of this cell population in animal reproduction in general, but also specifically in canine parturition. It remains to be further investigated, if the incomplete/inhomogeneous distribution of PTGS2 protein in the trophoblast cells of PUI samples (*n* = 3) was a coincidence or is important for uterine inertia itself or for its development.

Additionally, immunohistochemistry revealed the presence of PTGS2 protein in IP and UP myocytes, which had the strongest signals compared with all other immunopositive cell types. Whether the myometrium produces or contributes to PG, production relevant for parturient uterine contractions is contradictory in different species. Whereas in the rat, myometrial PTGS2 levels were found to increase with the onset of labour (Dong et al. 1996), myometrial PTGS2 mRNA and protein expression in women was reported to increase (Erkinheimo et al. 2000), decrease (Zuo et al. 1994), or remain unchanged (Moore et al. 1999; Myatt and Moore 1994; Sparey et al. 1999) at the onset of labour at term and preterm (Challis et al. 2002). By comparing myometrial staining intensity between OD and PC, our results indicate that in the dog, myometrial PTGS2 expression does not seem to be substantially modulated during parturition. A previous study in pregnant dogs found that *PTGS2* mRNA expression increased in UP tissue homogenates at prepartum luteolysis compared with early and mid-gestation (Kowalewski et al. 2010, Kowalewski et al. 2014), and the protein was strongly expressed in foetal trophoblasts indicating the importance of PTGS2 of placental origin during canine parturition. We could not verify significant differences in the overall myometrial staining score between OD and PUI. Therefore, a causative role of myometrial PTGS2 expression in PUI is not supported. Quantification of protein expression by Western blotting comparing myometrial PTGS2 expression in samples of PUI and OD bitches might confirm this observation.

Our finding of stronger PTGS2 protein signals in the longitudinal compared with the circular myometrial layer is new and may indicate different contractile abilities of the two myometrial layers. This has been shown by Gogny et al. using canine non-pregnant uterine strips in organ-bath and confirming a stronger response of the longitudinal compared with the circular myometrial layer to PGF2α (Gogny et al. 2010). In contrast to our findings, in pregnant rats, strongest myometrial staining was localized in the circular muscle layer, while only negligible staining was found in the longitudinal layer (Dong et al. 1996). It remains to be clarified in future studies if these findings reflect indeed species-specific differences. Additionally, tissue dissection and individual preparation of both myometrial layers for subsequent *PTGS2* mRNA and PTGS2 protein expression analysis by Western Blot seem to be advisable for future studies.

The protein expression of PGFS found in our samples was also in agreement with previous studies on canine pregnant uterus tissue using the same antibody (Gram et al. 2013; Kowalewski et al. 2010; Kowalewski et al. 2014). The only cell types that were positively staining for PGFS in all samples were the uterine luminal epithelial cells and the glandular epithelium of the superficial glands (glandular chambers of UP). Endometrial staining intensity was significantly higher in IP samples of PC than in OD (*p* = 0.0215), which is in line with the findings of Gram et al (2013) describing decreased PGFS protein expression at prepartum luteolysis compared with midgestation (days 35–40 of pregnancy) using western blotting in canine UP tissues (Gram et al. 2013). Our data indicate that PGFS protein expression in IP tissues decreases even further after prepartal luteolysis, when concomitant significant increases of PGFM are reported (Baan et al. 2008; Nohr et al. 1993). Additionally, no staining was found in the placental compartment. These observations further underline and confirm that PGFS is not the main source of canine prepartal PGF2α increase. Endometrial PGFS protein expression was not different between the PUI and OD groups, and thus, it is unlikely that PGFS played a role in the development of PUI.

## Conclusions

Our study on PTGS2, PGFS, and PTGFR expression brings new knowledge to the understanding of parturition-associated changes in the canine uterus (especially the myometrium) undergoing from quiescence to active labour (late pregnancy to parturition, i.e. PC to OD), and helps to identify possible differences between PUI and OD. Other than hypothesized, our results do not support the hypothesis of a role for PTGS2, PGFS, and PTGFR in the aetiology of PUI in the bitch. The observation of an overall stronger PTGS2 expression in the longitudinal myometrial layer compared with the circular layer is new, and further studies should be carried out to identify the physiological relevance of this finding. Our observation of undetectable PGFS protein expression in the placental compartment of PC and OD and reduced expression in IP tissue further supports the hypothesis that PGFS is not the source of the (peri-) parturient PGFM increase. Decreased *PTGFR* expression in UP samples of OD compared with PC bitches may be a consequence of previously reported large increases in peripheral PGFM concentrations (Baan et al. 2008; Baan et al. 2005; Concannon and Hansel 1977; Concannon et al. 1988; Concannon et al. 1989; Olsson et al. 2003) just before and during parturition, resulting in desensitization of the UP to the actions of PGF2α.

## Declarations

### Statement of the welfare of animals

All applicable national and institutional guidelines for the care and use of animals were followed. All procedures performed in studies involving animals were in accordance with the ethical standards of the institution at which the studies were conducted. Animal experimentation had been approved by the respective authorities (permit no. 2015-15-0201-00513, Dyreforsøgstilsynet Fødevarestyrelsen; permit no. ZH086/15, Cantonal Veterinary Office Zurich).
